# Resistance to a Rhabdovirus (VHSV) in Rainbow Trout: Identification of a Major QTL Related to Innate Mechanisms

**DOI:** 10.1371/journal.pone.0055302

**Published:** 2013-02-04

**Authors:** Eloi R. Verrier, Michel Dorson, Stéphane Mauger, Corinne Torhy, Céline Ciobotaru, Caroline Hervet, Nicolas Dechamp, Carine Genet, Pierre Boudinot, Edwige Quillet

**Affiliations:** 1 INRA, UMR 1313 Génétique Animale et Biologie Intégrative, Jouy-en-Josas, France; 2 INRA, UR892 Virologie et Immunologie Moléculaires, Jouy-en-Josas, France; 3 AgroParisTech, Paris, France; Temasek Life Sciences Laboratory, Singapore

## Abstract

Health control is a major issue in animal breeding and a better knowledge of the genetic bases of resistance to diseases is needed in farm animals including fish. The detection of quantitative trait loci (QTL) will help uncovering the genetic architecture of important traits and understanding the mechanisms involved in resistance to pathogens. We report here the detection of QTL for resistance to Viral Haemorrhagic Septicaemia Virus (VHSV), a major threat for European aquaculture industry. Two induced mitogynogenetic doubled haploid F2 rainbow trout (*Oncorhynchus mykiss*) families were used. These families combined the genome of susceptible and resistant F0 breeders and contained only fully homozygous individuals. For phenotyping, fish survival after an immersion challenge with the virus was recorded, as well as *in vitro* virus replication on fin explants. A bidirectional selective genotyping strategy identified seven QTL associated to survival. One of those QTL was significant at the genome-wide level and largely explained both survival and viral replication in fin explants in the different families of the design (up to 65% and 49% of phenotypic variance explained respectively). These results evidence the key role of innate defence in resistance to the virus and pave the way for the identification of the gene(s) responsible for resistance. The identification of a major QTL also opens appealing perspectives for selective breeding of fish with improved resistance.

## Introduction

Many lines of evidence in vertebrates have established that host genetics plays an important role in the relative susceptibility to infections to a range of pathogens including viruses. In human and monkey, the genetic restriction of HIV infection based on TRIM5a is one of the best examples [1]–[4]. In mice, a major locus also controls the resistance to leukemia viruses [5]. In farmed animals, genetic factors controlling resistance to several pathogens have been identified in pig, cow, sheep and chicken [6], and selective breeding is an appealing approach to improve the resistance to diseases in farm animals [7]–[11].

In fish, the existence of genetic variability associated with different levels of susceptibility to several diseases is well established. Significant heritabilities have been found for the resistance to various pathogens in marine and freshwater species [12], [13] and an increasing number of QTL (Quantitative Trait Loci) are being identified for resistance to a range of pathogens, including parasites [14], [15], bacteria [16], [17] and viruses [18]–[20]. These results provide a robust basis to promote resistance to diseases as a selective objective in the current aquaculture breeding programs [13], [21], [22].

Viral diseases currently constitute a major threat for aquaculture industry and, in some cases, for wild fish populations and fisheries [23]. Viral Haemorrhagic Septicemia virus (VHSV) has been reported in a number of wild and domestic marine or freshwater fish species [24], [25] and is considered as one of the most important fish viruses due to its great potential economic impact [26]. VHS is among the most studied fish viral diseases, especially in rainbow trout (*Oncorhynchus mykiss*) where it was originally described [27]. VHSV is a Novirhabdovirus, with a single strand RNA genome of negative polarity encoding five structural proteins (N, P, M, G and L) and the non structural NV protein specifically expressed in the Novirhabdovirus genus (reviewed in [28]).

Although considerable progress has been made in the understanding of finfish immune system, the respective importance of innate and adaptive immune mechanisms in the resistance to viruses is still poorly understood. The protective power of fish adaptive responses has been firmly established for different viruses including the Novirhabdoviurses IHNV and VHSV: efficient vaccines have been developed, which elicit specific B and T cell responses [29], [30]. In addition, the resistance to another virus - the Infectious Salmon Anaemia Virus (ISAV) - has been linked to the MHC, indicating a role for T cell immunity in the susceptibility to this virus [31]. However, innate and/or intrinsic factors are likely to play a key role in the genetic resistance to natural infections with rhabdoviruses. Comparing viral variants with variable virulences, or hosts with different genetic backgrounds, a suite of studies have shown that host mortality correlates with early virus load, faster replication enabling the virus to rapidly override the host defence [32]–[37]. Regarding VHSV, the correlation between genetic resistance and virus load in fin explants provided a good hint of the implication of such factors [32], [38]. The perfect correspondence between the resistance of clonal lines of rainbow trout and that of derived fibroblast cells *in* *vitro* definitely underlined the importance of innate/intrinsic components of immune response [37]. Additionally, the early and fast induction of IFN1 after infection was also involved in the resistance of some genotypes to the VHSV [37]. However, the general pathways and the key genes responsible for the variability of host resistance remain unknown.

Besides its potential to enhance selective breeding efficiency, QTL identification is also a first step toward the molecular dissection of complex phenotypes and toward the identification of associated causal mutations. In this study, we discovered several QTL for survival to VHSV after waterborne infection. We also established that the control of virus replication in fin explants and the fish survival to infection share common genetic bases, strongly suggesting that intrinsic/innate factors are responsible for much of the genetic variability of resistance to the virus. Our results pave the way to a better understanding of the mechanisms of viral response and the identification of the key genes involved and to new prospects for marker assisted selection of resistant fish.

## Materials and Methods

### Experimental Families and QTL Design

Several distinctive features of the trout model were considered to draw up the QTL mapping strategy. First, we took advantage of the fact that it is relatively easy to produce doubled haploid (DH) gynogenetic individuals by physical treatments (pressure or temperature) of ova previously fertilized with genetically inactivated milt. DH individuals contain two identical copies of the maternal chromosomes, *i.e.* they are fully homozygous, which facilitates the capture of extreme phenotypes/genotypes. In the present study, both F0 population and F2 mapping progeny were composed of DH individuals, allowing an accurate evaluation of their status and an increased power of QTL detection [39], [40]. Secondly, trout characteristics (large size family and low marginal cost of individual phenotypes for survival at infectious challenge) are propitious to a selective genotyping strategy. Selective genotyping has been shown to be highly cost effective with negligible disadvantages in term of power of detection provided a large population base can be easily phenotyped and that a sufficient number of individuals at each tail are genotyped to limit the risk of detection of false positive marker-trait associations [41], [42].

#### Selection of F0 breeders

Fish originated from the experimental outbred rainbow trout population maintained at the INRA experimental farm (Gournay-sur-Aronde, France) which is free of known viruses. Virological controls have been regularly performed on both healthy fish from each raceway (at least once a year) and on every group affected with unexpected mortality. No control was ever found positive. Hence, we believe that the original population was not subjected to any unintended selection by viruses including VHSV and IHNV.

Doubled haploid individuals (DH) were produced through mitogynogenetic reproduction of outbred females according to [43]. Some of the fry were hormonally sex-reversed to produce functional XX males. When adults, DH females and males were fin clipped and scored for the resistance status to VHSV using an *in* *vitro* test based on the correlation between the virus replication in excised fin tissue (VREFT value) and the survival of individual fish after an immersion challenge [32], [44]. The test is effective to select individuals transmitting resistance or susceptibility to the virus [38] without using direct inoculation that would definitely impair their use as breeders. Thirty nine DH individuals (F0 generation of the QTL design) were selected for respectively low (resistant, R) and high (susceptible, S) VREFT values and individuals with alternative resistance were pair-mated to produce F1 crosses. From offspring survival and contrast between VREFT values of the breeders, two F1 crosses (F98 and F00) were kept as future parents of the QTL families. The homozygous status of the corresponding F0 breeders (two females and two XX males) was checked using allelic variation at six microsatellite markers.

#### Production of doubled haploid (DH) mapping progeny

**Figure 1:**
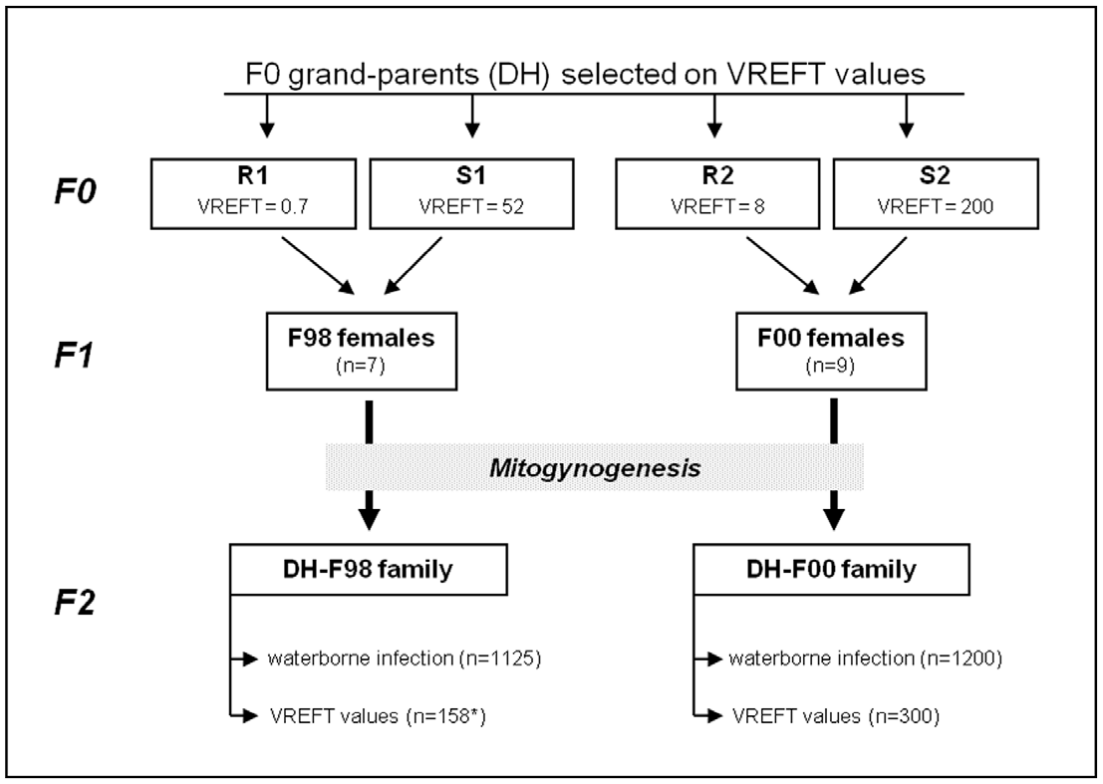
Production of experimental families and QTL design. DH: doubled haploid individuals (all homozygous), obtained by mitogynogenesis (fertilization of ova with UV irradiated sperm followed by inhibition of the first embryonic mitosis); VREFT values: viral replication in excised fin tissue (in pfu.mg^−1^); in each lineage, F1 females are isogenic, and their progeny were pooled into a single family. *:offspring used to measure VREFT values were produced one year later, from a single F98 female.

The family pedigree and experimental design are detailed in Figure 1. Because of their gynogenetic origin, F1 crosses were all females which were reproduced through a new generation of mitogynogenesis as previously described in [43]. The resulting offspring (DH) thus combine the two grand-parental genomes and have only one allelic type at every locus. In order to generate a sufficient number of progeny, several F98 and F00 individual females were used. As F1 females were isogenic (mating of two fully homozygous F0 individuals), DH progeny from females of the same F1 cross were further pooled into a single family (DH-F98 and DH-F00 families). Males homozygous for a dominant body colour mutation (golden phenotype) were used as milt donors for gynogenesis. The lack of golden fry in the progeny and of surviving fry in the haploid controls (no heat shock after fertilization with irradiated sperm) served as control of the efficiency of the irradiation process. Fertilized eggs were iodine disinfected and placed in units supplied with recirculated, dechlorinated tap water at 10°C constant (IERP, INRA, Jouy-en-Josas, France) where fish were reared until the infectious challenge.

### Phenotyping QTL Families for Resistance to VHSV

#### Survival at immersion challenge

Waterborne challenges were carried out on 4 to 5-months old juveniles using the VHSV strain 07–71 (serotype 1) as described in [32].

VHS virus strain 07–71 was isolated in 1971 from diseased fish sampled in a trout farm in Normandy (France) [45]. This virus belongs to the type 1 serotype originally described in Denmark. This strain has been propagated since then in epithelioma papulosum cyprinid (EPC) cell line, which provides high titers. Virus titers were determined as pfu.ml^−1^ using the plaque assay method as described in [37]. For the challenges analysed in this work, a large virus production was titered, aliquoted and kept at −70°C until use. Regular titering provided values consistent with the initial assessment, indicating that the different experiments were performed in similar conditions (*i.e.* with the same number of pfu per ml). Virus suspensions were thawed just before infection and kept in ice until use. For each DH family, a little over one thousand fish were distributed into 10 l aquaria (125 fish into 9 aquaria and 120 fish into 10 aquaria for DH-98 and DH-00 respectively) and were infected by incubation for 2 h in a 5×10^4^ pfu.ml^−1^ virus suspension in static water with vigorous aeration. This protocol regularly leads to 80–90% mortality in the INRA outbred rainbow trout population. Water supply was set up again after exposure to the virus. Mortality was monitored during 34 days for F98 and 58 days for F00.

Dead fish were removed twice a day. At the end of the period of survey, surviving fish were sacrificed (lethal anaesthesia with 2-phenoxyethanol). All individuals were immersed in absolute ethanol for further DNA extraction. Although not all dead fish could be used for virus re-isolation, we could re-isolate the virus regularly from moribund infected animals during the project. In addition, we did not observe any mortality associated to the typical clinical signs of the VHS in controls.

#### Measurement of VREFT values

Remaining uninfected DH-F00 progeny were kept in the controlled rearing unit for further growth (Figure 1). When they weighted about 35 g, 300 fish were anaesthetized and rayed fin (anal and/or pelvic) was clipped and processed as described in [46] to perform a measure of viral replication in excised fin tissue (VREFT). Briefly, fin explants (mean weight: 74 mg) were infected by immersion for 1 h in 2 ml of Stoker’s medium containing 2×10^5^ VHSV pfu.ml^−1^, then rinsed and incubated for 3 days at 14°C. The virus titration (pfu.mg^−1^ of fin explant) was then measured using the number of plaques recorded on EPC monolayers after inoculation with a serial 10-fold dilutions of ground explants. Because of a problem in the facilities, DH-F98 fry were no more available for VREFT measure. Subsequently, one F98 female was used the next spawning season to produce a genetically identical DH offspring. Fertilization and rearing procedures were as previously described. Fin explants (mean weight: 34 mg) were sampled on 158 individuals (11 g body weight) and processed as for DH-F00. A piece of fin of every sampled fish was kept in absolute ethanol for further genotyping.

### Microsatellite Genotyping

As indicated above, a bidirectional selective genotyping approach was applied to search for QTL associated to survival after waterborne infection. Within each family, highly susceptible and highly resistant individuals (‘tails’) were selected. The susceptible tail was composed of the 10% most susceptible fish (first to die) from every aquarium. In DH-F98, the resistant tail was composed of survivors (n = 95, 8% of the challenged fish). In DH-F00, a random sample of survivors was taken in order to limit the size of the resistant tail to 10% of the population (120 individuals). Thus, the fraction of genotyped individuals relative to the whole population was around 20%, ensuring a reasonable power of QTL detection considering the whole size of the population and the marker density [42], [47], [48]. F0 grand-parents and F1 females were also genotyped. The genome scan was performed with 131 and 142 polymorphic microsatellite markers including 25 and 18 duplicated markers for DH-F98 and DH-F00 respectively (Supporting information Table S1 and Table S2) evenly distributed along the INRA reference map [49], [50]. Average overall spacing between markers was 22.1 cM (DH-F98) and 20.4 cM (DH-F00) (2 to 10 markers per linkage group). Search for VREFT-associated QTL was focused on the regions where a significant QTL for survival was detected. Subsequently, VREFT-phenotyped progeny were genotyped for a set of microsatellites (4 to 8) in each linkage group of interest (Supporting information File S1).

DNA extraction and genotyping methods for microsatellite markers were described in [49], [50]. Linkage groups were named according to [50]. A linkage map was rebuilt for the families of the design using all genotyped individuals and CarthaGène software [51], [52].

### Association Studies and QTL Detection

In the selective genotyping approach (survival-associated QTL), marker-trait association was inferred by testing difference in marker allele frequencies among the two population tails. The Pearson χ^2^ test (1 dl) was performed at every marker used for the genome scan to search for association between the issue of infectious challenge (dead/surviving) and the genotype (R *vs* S grand-parental allele). Bonferroni correction was considered for multiple tests at the chromosome-wide level.

Detection for VREFT-associated QTL was performed using QTLMap software [53]. An interval mapping method described by Elsen *et al.* [54] was applied, scanning for QTL every 1 cM in the genome. To take into account the uniparental origin of the DH families, they were considered as full-sib families where each fish was assigned a virtual unknown parent different for every individual. For each F1 dam, QTL effects were estimated as the allelic substitution effects. The hypotheses of the presence of one QTL (H1) *vs* no QTL (H0) at one location were compared using an approximate likelihood ratio test (LRT) [55]. For each linkage group, the empirical distribution of LRT was obtained from 10000 simulations under the null hypothesis, with a trait heritability fixed to 0.5. Thresholds of H0 rejection at the chromosome-wide level were then estimated with the method described by HARREL and DAVIS [56]. The 95% confidence QTL support intervals were obtained using the “one LOD drop-off method” [41]. Log-transformed VREFT values were used to meet the assumption of normality.

One limitation of selective genotyping is the difficulty to estimate unbiased QTL genetic effects [42]. In order to estimate the effect of relevant survival-associated QTL more precisely, a comprehensive analysis was carried out including all fish in a subset of three aquaria per DH family. A whole post challenge life-time dataset was built. Individual data were the surviving status (dead or alive at the end of the challenge) and the time to death (TTD, in days after infection) of each fish that died. Surviving fish corresponded to ‘censored’ observations, *i.e.* that the expected event (death) was not recorded during the observation period. Fish not genotyped yet (dead after the first 10% and, for DH-F00, survivors not previously sampled) were genotyped for the markers of the target region. Data were pre-corrected for fixed effect (aquarium). QTL detection was performed with QTLMap software using an interval mapping method for survival phenotypes (non-normal distribution and presence of censored data) based on Cox model and described by [57]. Pedigree design was encoded as previously described and methods to test the presence of QTL and to determine thresholds were the same.

The QTL effects (% of phenotypic variance explained) were estimated by testing the within family effect of the alternative alleles at the marker closest to the QTL location (ANOVA with SAS software). QTL effect on survival was estimated using TTD, assuming a Gaussian distribution and fixing the maximum TTD at day 28 in DH-F98 (day of the last mortality recorded in the family). The same limit was chosen in DH-00 to facilitate comparison and to avoid a too high dispersion of TTD values.

#### Ethics Statements

Animals were handled in accordance with the national and European guidelines and regulations on laboratory animals care in force at the time of the experiments. Animal work was approved by the French Veterinary Services (personal authorizations numbers 78-67 and 32–57 for Edwige QUILLET and Michel DORSON respectively). During the infectious challenge, water quality was regularly monitored. Fish were fed and controlled twice a day. Dead fish were systematically removed as well as fish obviously suffering (abnormal swimming behaviour) that were euthanized using lethal anaesthesia.

## Results

### Production of DH Families from F0 Grandparents Selected for Opposite Resistance or Susceptibility to VHSV

In order to increase the probability of detecting QTL, F1 parents of the mapping progeny were generated by crossing F0 individuals with opposite expected resistance to the virus. To this purpose, VREFT values were assessed from a number of DH progeny from females randomly sampled in the original outbred population, and used as a criterion for choice of four F0 breeders. VREFT values in the initial F0 population (143 DH individuals) ranged from 0 to 5454 pfu.mg^1^, a range slightly narrower than previously observed in standard individuals from the same population [32]. VREFT values of the 39 DH individuals retained to produce the parental F1 crosses spanned the observed variability (0 to 4762 pfu.mg^1^). Two critical issues affected the production of F2 families: many F1 crosses failed because of the poor reproductive performance of DH (fully inbred) individuals [58] and the deleterious impact of treatments used to induce mitogynogenesis with ova of F1 females often yielding low survival in F2 offspring. Finally, two F2 families with a sufficient number of DH fry could be kept (DH-F98 and DH-F00). The four F0 individuals were progeny of four different females from the original population.

The contrast between the VREFT values of the corresponding F0 grandparents was lower than expected (0.7 *vs* 52 pfu.mg^1^ for DH-F98 and 8 *vs* 200 pfu.mg^1^ for DH-F00). Yet, our previous results had shown that VREFT values below 10 pfu.mg^1^ are associated with high survival, while susceptibility increases dramatically for values above that threshold [32]. Therefore, we classified the two F0 individuals with low VREFT values (0.7 and 8 pfu.mg^1^) as resistant and the two F0 ones with higher VREFT values (52 and 200 pfu.mg^1^) as susceptible (Figure 1).

### Genetic Status and Average Performances of DH Offspring

Average survival of DH progeny was much higher than usually recorded in experiments where mitogynogenesis is induced from outbred females (Table 1). The use of fully homozygous DH grandparents likely contributed to this good yield, by reducing the number of segregating lethal and deleterious alleles. In DH-98 family, a small proportion of wild type surviving fry was recorded in the haploid controls (Table 1). Such fry are usually considered as the result of spontaneously unreduced ova. Indeed, they were all normally pigmented (no golden phenotype), indicating that the sperm irradiation process had been successful. In this family, genotyping of juveniles few months later revealed an unexpectedly high proportion of non-fully homozygous individuals (overall proportion of 10.5%). As in the haploid control, those individuals were normally pigmented. Moreover, they carried only the expected maternal F1 alleles, which supported the hypothesis that F98 females produced a small proportion of spontaneously unreduced ova. The higher proportion of resulting individuals in several months old juveniles than in larvae is likely due to a higher mortality at early life stages in the ‘true’ homozygous DH than in those partially heterozygous individuals.

**Table 1:** Main characteristics of DH families until infectious challenge.

Family	% H^a^	% DH embryos^b^	% DH juveniles^c^	Body weight
DH-F98	1.5	41.0	37.8	1.3 g
DH-F00	∼0	32.4	36.4	1.6 g

a survival in the haploid control group (H) at the end of yolk resorption (% ova fertilized with irradiated sperm without diploidization treatment). In DH-F98∶49 wild pigmented type surviving fry out of 3230 fertilized ova. In DH-F00: no wild type surviving fry and one single surviving fry with golden phenotype out of 1900 fertilized ova.

b normal eyed embryos in DH progeny (% fertilized ova).

c 4–5 month-old DH juveniles (% eyed embryos).

In contrast, no wild type individual survived in the DH-F00 haploid group. Further genotyping of juveniles detected only one non homozygous individual, confirming that frequency of unreduced ova was very low in F00 females. One single fry with golden phenotype (indicating fecundation with intact sperm) was recorded in the haploid control. The origin of this unique golden fry remains intriguing, as other fish in this family were all normally pigmented and carried only the expected maternal alleles, confirming the overall efficiency of the irradiation process. All non homozygous individuals were discarded from further analyses (survival and VREFT measurement).

**Figure 2:**
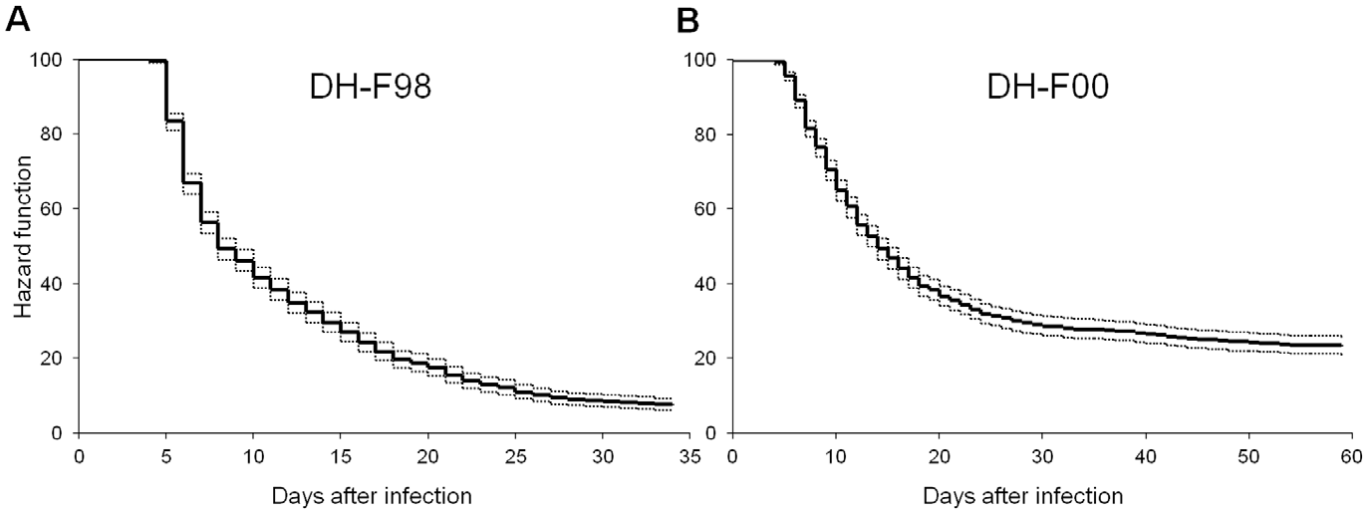
Cumulative survival curves after waterborne infection of the two DH families used for selective genotyping. Kaplan-Meier estimation of survival functions after infectious challenge for the two DH families. Fish were infected by incubation for 2 h with the VHSV strain 07–71. All fish, including heterozygous individuals detected after genotyping of the phenotypic tails, were conserved to draw the curve. (A) Hazard function of DH-F98 progeny (1125 fish challenged, 9 aquaria). (B) Hazard function of DH-F00 progeny (1200 fish challenged, 10 aquaria).

To identify survival-associated QTL, an immersion challenge was carried out when fish weighted around 1–2 g. The cumulative survival curves were quite typical of development of VHSV infection after waterborne challenge (Figure 2). Final survival was 8% and 23% in DH-F98 and DH-F00 respectively. To identify QTL for viral production on fin explants, VREFT values were determined for 133 DH-F98 individuals and 300 DH-F00 individuals. Mean values were 256 and 792 pfu.mg^1^ respectively. The corresponding ranges were [2.2–5,529] and [0.9–36,293] pfu.mg^1^.

### Several Survival–associated QTL Identified by Genotyping Individuals with Extreme Phenotypes

**Figure 3:**
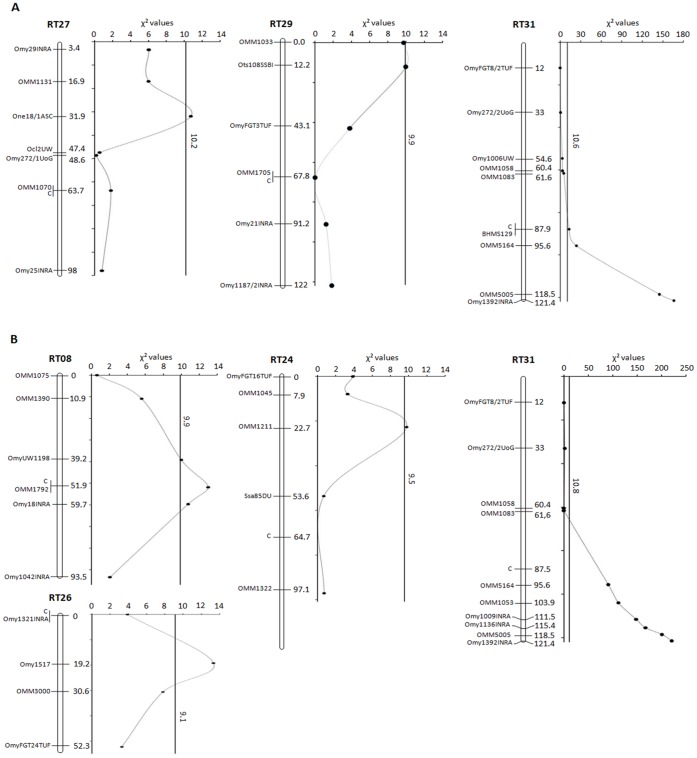
Survival-associated QTL detected in DH-F98 and DH-F00 families after VHSV waterborne infection using a bidirectional selective genotyping strategy. The figure shows significant QTL identified in DH-F98 (A) and DH-F00 families (B). In each family, about 20% of the entire population (1125 and 1200 fish respectively) were genotyped, *i.e*. about 10% extremely susceptible individuals (first to die) and 8% (DH-F98) to 10% (DH-F00) resistant fish (survivors) according to the survival at the end of the challenge. Only linkage groups with significant QTL are shown. Linkage groups are labelled according to (Guyomard *et* *al.* 2012). C indicates the position of centromere. Marker positions are given on the linkage groups rebuilt for each family from all genotyped individuals. At every marker position, a P-value <0.01 at the chromosome-wide level (χ^2^-test with Bonferroni correction) was taken as the significance threshold. For each linkage group, the black line indicates the significance threshold χ^2^ value at the chromosome-wide level. The QTL on RT31 was also significant at the genome-wide level.

Results of association tests between markers and fish survival are shown in Figure 3, Table S1 and Table S2. Seven significant survival-associated QTL were detected (P<0.01 at the chromosome-wide level) on six different linkage groups. Three and four QTL were identified in DH-F98 and in DH-F00 family respectively. One QTL only was mapped in both crosses, in the telomeric region of linkage group RT31. This common QTL was highly significant: almost all surviving fish carried the allele of the R grandparent at the closest locus. Effects of other QTL were consistent with the known lineage in the DH-F00 family (higher survival associated with the R grandparent allele) while in the DH-F98 family, the resistant offspring carried preferentially the S grandparent alleles at the two other QTL loci segregating in the family. In fact, most of the resistance to VHSV infection was explained by the same genomic region in both families. Few additional regions were also involved, but with effects more disparate and of lesser magnitude.

**Figure 4:**
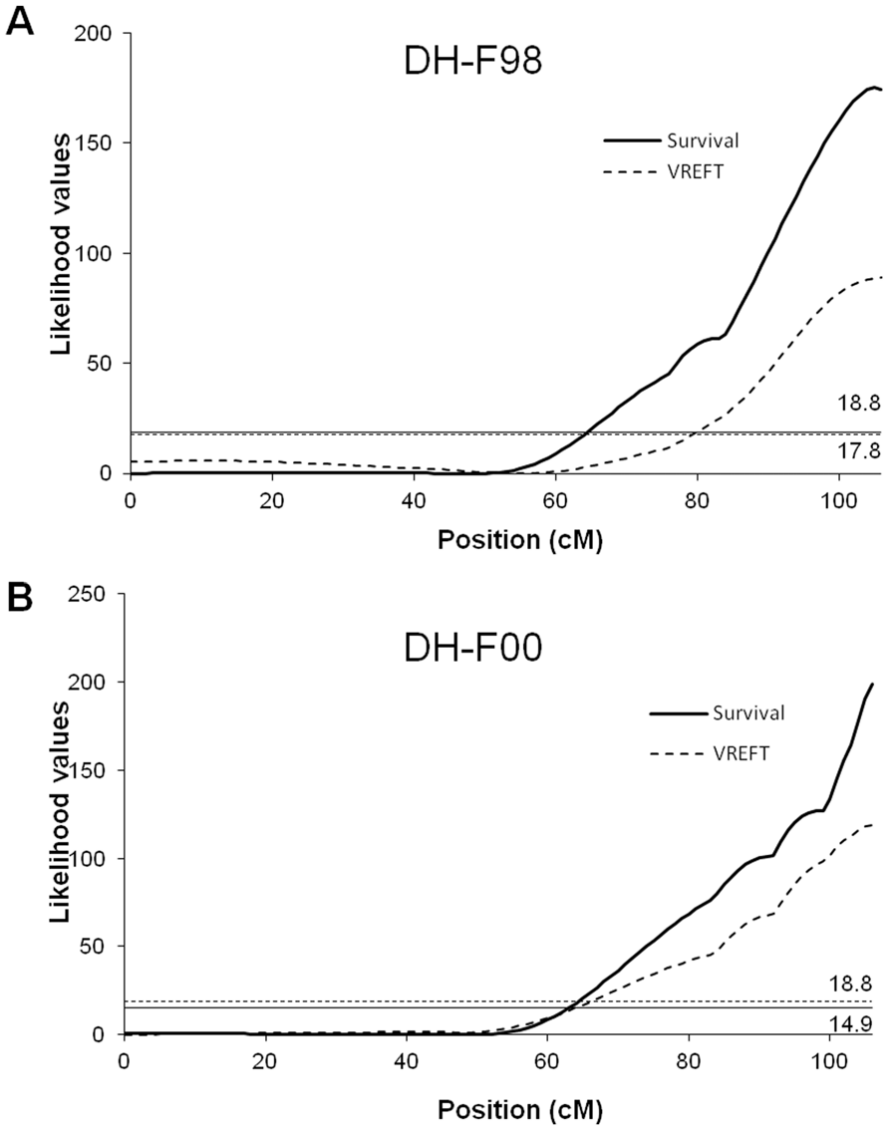
Likelihood ratio profiles for survival and VREFT values associated QTL on linkage group RT31 in DH-F98 and DH-F00 families. Analyses for the survival-associated QTL were performed with the complete post challenge life-time dataset (3 aquaria per family, *i.e.* 354 fish in HD-F98 family and 360 fish in HD-F00 family, heterozygous individuals excluded). For VREFT-associated QTL, 133 and 300 fish respectively were analysed in the two families. A total of 8 and 10 markers located on RT31 were genotyped in DH-F98 and DH-F00 respectively. Marker names and positions are available on supplementary Table 1S. Likelihood values were obtained using the QTLMap software. Likelihood thresholds for significant QTL (P<0.01 at the genome-wide level) were determined from LRT distribution of 10000 simulations under the null hypothesis (no QTL). Survival-associated QTL locate at 105 and 106 cM in DH-F98 and DH-F00 respectively. VREFT-associated QTL locate at 106 cM in both families (confidence intervals estimated at about 1 cM in length).

In order to refine the estimation of the major QTL effect (RT31), an analysis including life-time data throughout the course of the infectious challenge was performed in a subset of three aquaria of 120–125 fish per family. Individuals were genotyped for the same 10 microsatellites of the RT31 linkage group. Results were very consistent with the results of the selective approach (Figure 4), the QTL being again highly significant in both families (P<0.01 at the genome-wide level), and located at the same position, at the end of the linkage group, close to OMM5005. Because of this distal position, no clear support interval could be established for the QTL. However, according to LRT values, interval corresponding to 1 cM around QTL position could be established in DH-F98 family. Only the first quote could be assessed in DH-F00 (1 cM before the maximum) because no decrease of LRT values was observed after QTL position.

**Figure 5:**
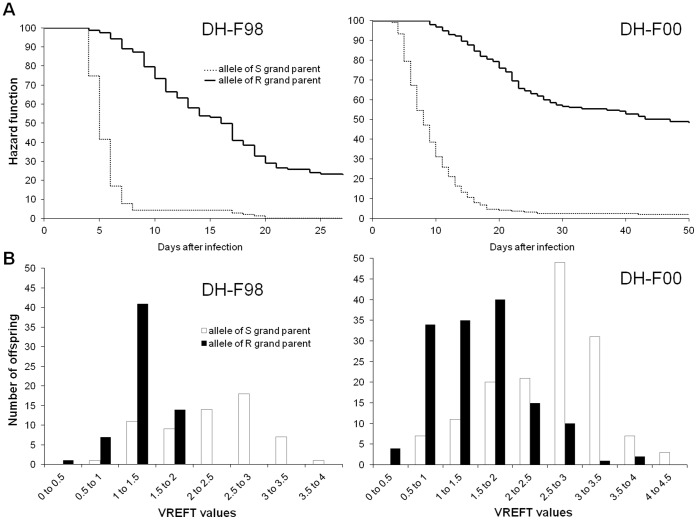
Effect of allelic status at OMM5005 marker (RT31) on cumulative survival curves during infectious challenge and distribution of VREFT values in the two DH families. (A) Cumulative hazard functions (Kaplan-Meier estimator) for the alternative R/S alleles. (B) Frequency distribution of VREFT values in the two families for the alternative R/S alleles. VREFT values (pfu.mg^−1^) are log-transformed.

The effect of the QTL (RT31) on survival was estimated from the Cox regression model used in QTLMap by the relative risks according to the QTL allelic status. Risks provide time-independent relative estimators of the susceptibility of different groups to the virus. Taking the risk of fish homozygous for the R allele at the QTL as reference (risk fixed at 1), the risk associated to homozygous status for the alternative S allele was 7.0 and 7.3 in DH-F98 and DH-F00 families, respectively, which was significantly higher than the reference risk (χ2-tests (1 df) significant at P<0.01). The cumulative hazard functions (Kaplan-Meier estimators) according to the allelic status at marker OMM5005 clearly illustrate this strong difference (Figure 5A). Using TTD (time to death) as the resistance trait, the QTL explained 44% to 65% of the phenotypic variance (Table 2).

**Table 2:** Performances (means and standard deviations) according to the alternative alleles at OMM5005 marker and percentage of the phenotypic variance explained for time to death (TTD) and viral replication in fin explants (VREFT).

Trait	Family	Values		*R* ^2^
		R allele offspring	S allele offspring	
TTD	DH-F98	16.6±7.6	6.1±3.6	0.44
	DH-F00	24.2±5.7	9.5±5.1	0.65
VREFT	DH-F98	1.3±0.3	2.3±0.7	0.49
	DH-F00	1.6±0.7	2.6±0.8	0.33

### The Major Survival-associated QTL Controlled Viral Replication in Excised Fin Tissues

Our previous studies with the VREFT test and fibroblast cells from clonal trout lines have raised the hypothesis that innate/intrinsic defence mechanisms may largely determine the outcome of infection. To further investigate the genetic link between fish survival and viral proliferation on fin explants, we tested whether the genomic regions that control survival after infection were also involved in the variability of the VREFT phenotype. Linkage associations for VREFT values were performed on the target linkage groups (RT27, RT29, RT31 in DH-98 and RT08, RT24, RT26 and RT31 in DH-00). They revealed only one significant QTL (P<0.01 at the genome-wide level) in both families, on RT31 at the same position than the survival-associated major QTL (Figures 4 and 5B). This QTL explained 33 to 49% of the VREFT phenotypic variance (Table 2). No other QTL could be identified on any of the six other linkage groups, even when a correction for a fixed effect corresponding to the allelic effect of the major QTL on RT31 was applied prior analysis.

### The Major Survival-associated QTL was Detected in Two Additional Families

Results of the two DH families suggested that a common major QTL plays a pivotal role in the resistance/susceptibility to VHSV in different genetic backgrounds. To provide additional support to this result, we searched for the survival-associated QTL in two additional DH families. The two families (DH-F01 ad DH-F21) had been produced according to the same procedure as DH-F98 and DH-F00 (F2 progeny from breeders selected for VREFT values) but were not used in the first phase of the QTL detection because of a shortage of surviving juveniles. In addition to the selection on VREFT values, the additional F0 breeders were chosen to have no relatedness with the ones previously tested, with the exception of one for which the pedigree was unknown. The same selective strategy (early dead *vs* survivors) was performed and the two tail-groups were genotyped at the closest survival-associated QTL marker (OMM5005). Alternative alleles were almost fixed in the two extreme phenotypes accordingly to the expected grand-parental origin (Table 3), which confirmed the key role of this major QTL in the variability of resistance to the virus.

**Table 3:** Characteristics of additional DH families and marker-trait associations at OMM5005 in extreme phenotypes (early dead/surviving).

Family	VREFT of F0 grandparents^a^		Number challenged	Survival at challenge	Dead		Surviving		?^2^value^c^
	Susceptible	Resistant			S b	R b	S	R	
DH-F01	2605	0.75	375	35.5%	82	3	2	68	131.8
DH-F21	667	0.25	390	24.4%	85	0	1	78	156.1

a (pfu.mg^−1^).

b R: allele from the resistant grandparent; S: allele from the susceptible grandparent.

c the threshold value for χ^2^-test (1 df) at P<0.01 at the chromosome level is 10.8.

Each family was challenged in 3 aquaria. In every aquarium, the first 30 fish to die and up to 30 survivors were sampled, *i.e.* a sample of 90 dead *vs* 72 and 80 survivors in DH-F01 and DH-F21 respectively. Thirteen fish had missing genotype.

## Discussion

In this work, we have identified a major QTL that consistently explains a large part of resistance to a rhabdovirus waterborne infection in rainbow trout, a fish species important for aquaculture. The same QTL controls both the survival of juvenile fish after VHSV infection and the virus replication on fin explants. Thus, these two traits are governed by the same genomic region, which strengthens the idea of a key role for intrinsic/innate defence in the resistance to the virus [32], [35], [37]. The critical role of a major QTL in the resistance to VHS does not exclude a contribution of other defence mechanisms. In particular, adaptive immunity might be especially important at later stages of the life of the fish.

Additionally, the effect of QTL was assessed in an experimental population having a unique genetic structure (fully homozygous doubled haploid) and should be confirmed in standard outbred trout populations.

In total, seven QTL for survival were mapped on six different linkage groups, but only one major QTL was observed in the different genetic backgrounds. Other QTLs were cross-specific, *i.e.* mapped in a single family. Altogether, it seems that they play smaller roles in survival, even in the genetic backgrounds where they were detected. However, the selective strategy was not suitable to precisely assess their contribution to resistance since they may have been partly masked by the effect of the major QTL. Moreover, because of the medium density of the genome coverage for QTL detection, additional QTL with smaller effect may have been missed. These minor QTL may help to identify the sequence of genes involved in the resistance at the whole organism level, and could be critical in specific contexts. Interestingly, the closest marker to the RT29 secondary QTL in DH-98 family (Ots108SSBI) was mapped very close to one Mx gene according to [50]. Mx genes belong to the interferon stimulated genes (ISGs) involved in innate immune response to viruses (for a review, [59]). Notably, polymorphism of Mx sequences has been associated with variation of resistance to another rhabdovirus - the IHNV - in rainbow trout [60]. As previously mentioned, the allele effect at Ots108SSBI was contrary to the one expected from lineage, *i.e.* the S grandparent transmitted the resistance allele. The favorable effect of the S allele on resistance (departure from a 1∶1 allelic ratio significant at P = 0.002) was recorded in survivors also having the favorable allele at the major QTL, but not in dead individuals, suggesting that a second defensive line involving Mx might follow the effect of the major QTL and confer a higher level of resistance.

It is noteworthy that none of the secondary survival-associated QTLs was retrieved as VREFT-associated QTL, and that only one VREFT-associated QTL was detected. This may be due to a lower power of detection compared to survival QTL, *e.g.* due to insufficient number of samples (as in DH-98, where only 133 offspring were available) or to imprecise phenotyping. Indeed, using VREFT values may misclassify some individuals (‘false’ resistant fish, [12], [32]). More generally, viral resistance phenotypes are difficult to assess as many non genetic factors modulate the virus entry or subsequent viral replication [61]. This is well illustrated by the fish-to-fish dispersion of the time to death among individuals belonging to the same clonal trout line despite their genetic homogeneity [44]. In the same line, one cannot exclude that a complete genome scan for VREFT phenotypes would have detected some QTL exclusively controlling viral replication without correlated effect on fish survival. Viral replication in fin explants and survival might rely on distinct mechanisms: indeed, the major QTL could contain several different genes playing different roles in the resistance and viral replication in fin explants, respectively. However, the detection of a major QTL common to both traits may also reflect the existence of a mechanism governing virus replication, which in turn determines further survival of fish. This would be well consistent with studies underlying the importance of early replication kinetics in the outcome of the viral infection. Hence, the major QTL on RT31 is a very attractive candidate for further fine mapping and identification of the causative gene(s). Several lines of evidence suggest that the intrinsic/innate mechanisms behind resistance are likely to act in the very first steps of the rhabdovirus infection [32], [35], [37]. Thus, relevant candidate genes should likely involve antiviral factors, including moderators of virus entry, replication or production.

The detection of a unique QTL for viral replication could suggest a single-step underlying mechanism, like a receptor-type mechanism. Little is known about potential VHSV receptors. It has been shown that fibronectin could mediate the cell entry of the virus [62]. However, there is still no information about the possible location of any trout fibronectin gene within the QTL region.

Post-entry mechanisms, for example variations of the kinetics of gene expression involved in innate immune response to infection could also be involved. Indeed, a number of such genes are overexpressed after VHSV infection, like *ifnφ, viperin/vig-1*, *isg15* or genes involved in NF-κB signal transduction [63]–[65]. Our previous work using clonal lines of rainbow trout and derived fibroblastic cell lines [37] indicated that, at least for one line, resistance is associated to a very early induction of functional IFN-φ1 (within the first hours after infection). In this case, genes acting upstream the IFN production – such as virus sensors or signalling molecules upstream IFN - would be the pivotal factors of the resistance.

In this context, TLRs (Toll-Like Receptors) constitute relevant candidates. TLRs are transmembrane proteins acting as Pattern Recognition Receptors (PRRs), recognizing pathogen structures and inducing innate immune responses (for a review, [66]). TLR7 and TLR8 recognize single-stranded viral RNA and are located in the trout genome close to the OMM5005 marker on RT31 [67], promoting their interest as possible candidate genes in the major QTL we detected.

Interestingly, the OMM5005 marker itself is located close to a gene encoding the protein SPRYD7 since it was derived from the EST 1RT90I19. SPRYD7 seems to be conserved in vertebrates, but its function remains unknown. However, the SPRY domain is found in many TRIM proteins involved in antiviral defence [68], [69]. Moreover, the gene *trim13* that is involved in autophagy regulation and cellular stress in mammals [70] is closely linked to *spryd7* in different fish and other vertebrate genomes.

Further investigations are needed to identify causative genes. The rapid development of genomic resources in rainbow trout, including SNP makers for high throughput genotyping [71], [72] and the next release of the genome sequence [73], [74] will be most helpful. In salmonids, the recombination patterns during meiosis are characterized by large sex differences, leading to sex-specific linkage maps longer in females. Regional differences are also observed, with usually higher recombination rates in centromeric regions in female maps and in telomeric regions in male maps [75], [76]. During this study, we failed in extending the map around the QTL, because there was almost no recombination at the end of the chromosome arm in the female device we used. Although our results indicate a relatively tight QTL support interval (a few cM), we suspect from male maps [72] that the actual interval is much larger, which may complicate the identification of relevant positional candidate genes. Using a male device might be an alternative for future investigations aiming at bounding the QTL and refining its position.

Another example of a major QTL governing resistance to a virus in fish has been recorded in salmonids, *i.e.* the QTL for resistance to IPNV (Infectious Pancreatic Necrosis Virus), in Atlantic salmon (*Salmo salar*). This QTL also explains a very large proportion of the genetic variance of resistance in Scottish and Norwegian farmed populations [19], [20]. Fine mapping of such QTLs and identification of underlying causative genes represent major challenges for the next few years. These findings will provide an in depth understanding of the basis of resistance variations in teleost fish and beyond, and will help the development of efficient strategies of health control in farmed fish.

Fish selective breeding is a very dynamic industry. However, disease resistances are traits usually difficult to select for in commercial breeding programs. For such traits, marker assisted selection may be particularly advantageous over conventional breeding methods. In this respect, the identification of a major QTL for resistance to VHSV, a major threat for rainbow trout industry, opens attractive prospects.

## Supporting Information

Table S1 **Markers used for detection of survival-associated QTL and results of association tests for family DH-F98.**
(PDF)

Table S2 **Markers used for detection of survival-associated QTL and results of association tests for family DH-F00.**
(PDF)

File S1 **Markers used to refine the effect of the major survival-associated QTL and for detection of VREFT-associated QTL in the two DH families.**
(XLS)
