# Supplementary material for: Resistance to a Rhabdovirus (VHSV) in Rainbow Trout: Identification of a Major QTL Related to Innate Mechanisms
Source: PLoS One. 2013 Feb 4;8(2):e55302. doi: 10.1371/journal.pone.0055302 (PMC3563530; doi:10.1371/journal.pone.0055302)
Supplement: Table S1 — Markers used for detection of survival-associated QTL and results of association tests for family DH-F98. (PDF) [file pone.0055302.s001.pdf]

TABLE S1

List of microsatellite markers used for detection of survival-associated QTL (DH-F98)

Linkage Group	Marker	Position	Accession number	χ^2
RT1	OmyFGT19/1TUF	7.6	Sakamoto [a]	0.08
	Omy1200INRA	24.2	BV681488	0.015
	Omy1326INRA	54.2	BV681457	0.995
RT2	Str1BFRO/1	34.9	U90327	1.211
	OmyFGT18/1TUF	111.2	Sakamoto [a]	0.822
	OMM1200	111.2	AF469985	0.343
	OMM1218/1	115.4	AF469991	0.293
	OMM1206/1	117.1	AF470000	0.022
RT3	Omi161TUF	12.3	AB105853	0.686
	OMM1230	37.2	AF470010	0
	OGO2/1UW	72.1	AF007827	0.001
	Omy1097INRA	104.3	BV681357	1.82
RT4_25	OMM1193	15.6	AF469978	0.037
	Ogo2/2UW	100.1	AF009794	0.57
	OMM1054	132.8	AF352739	1.151
RT5	OMM1728	26.4	BV212208	0.207
	OMM1032	60.3	AF352737	0.46
RT6	OMM1231	9.2	AF470011	0.51
	Omy1143INRA	36.7	BV681517	0.01
	OMM1776	120.2	BV212244	0.82
RT7	Omy207UoG	29.7	O'Connell [b]	0.47
	OMM1351	66.1	G73581	0.53
	One1/2ASC	96.9	U56699	0.9
RT8	OMM1075	0	AF352746	0.23
	OMM1792	37.1	BV212253	0.45
	Omy18INRA	59.4	Pr009689144.1	0.08
RT9	OMM1206/2	0.8	AF469991	0.02
	OMM1218/2	4.1	AF470000	0
	OMM1200/2	4.1	AF469985	0.03
	OmyFGT18/2TUF	5.2	Sakamoto [a]	0.09
	OmyFGT28TUF	24.5	Sakamoto [a]	0.31
	OMM1450	58.7	BV079594	0.25
	OMM1130	84.6	AF375031	0.08
	OMM1096	98.8	AF352762	0.41
RT10	OMM1179	0	AF469966	0.03
	One10ASC	17.8	U56710	1.01
	OmyRGT2TUF	37.2	AB087587	3.77
	OMM1753	67.7	BV212227	4.16
	OMM5186	85.5	BV212286	2.48

	OMM1202/1	104.3	AF469987	4.02
RT11	Ocl1UW	12.4	AF028694	4.83
	Omy7DIAS	15.7	AF239043	6.89
	SSOSL32/1	42.3	Z69642	0.91
	Omy23INRA	52.9	Pr009689146.1	0.34
	OMM1004	52.9	AF375008	0.26
RT12	OMM5233	15.7	CA349039	0
	OMM1030	45.4	AF375012	1.98
	OMM3059	78	BV078095	0
	OMM1381	95.1	BV078067	1.08
RT13	OMM1216	6.1	AF469998	0.06
	Omy1479INRA	45.2	BV686475	0.69
RT14	OMM1241	1.7	AF470021	0
	Omy1214INRA	23.6	BV681478	2.87
	SSa14DU	54.1	McConnell [c]	0.2
	Omy120/2INRA	100.7	Pr009689154.1	0.17
	Omy4/1INRA	110.9	Pr009689136.1	0.16
RT15	OMM1455	17.2	BV079599	0.35
	Ots1BML	53.2	AF107029	0
	Omy14INRA	83.6	Pr009689141.1	0.26
	Omy1383INRA	98.2	BV681442	3.65
RT16	OMM1101	8.6	AF352765	3.66
	Omy1038INRA	23.1	BV681522	2.31
	OMM1013	59.5	AF346674	0.02
	OMM1156	90.7	AY039638	0.67
	OMM1403	109.2	BV078088	0.57
RT17	OMM1076	15.6	AF352747	0.12
	OtsG422	49.2	AF393197	0.94
	OMM1775	87.4	BV212243	0.04
RT18	Omy1DIAS	46	U93270	0.03
	OMM1311	50.2	G73551	0.28
	Omy335UOG	67.3	Jackson [d]	2.02
RT19	Ocl8UW	0	AF028697	0.04
	OMM1025	40.2	AF346682	0.01
	OMM1313	60.7	G73553	0.02
	One3ASC	98.8	U56701	0.1
RT20	OMM1050	11.8	AF346694	0.06
	Omy8DIAS	48.5	AF090390	9.23
	Omi102/1TUF	78.8	AB105844	0.2
	Omy120/1INRA	95.7	Pr009689154.1	1.99
	Omy4/1INRA	103.5	Pr009689136.1	0.29
RT21	OmyFGT2TUF	21.8	Sakamoto [a]	0.18
	OmyFGT27TUF	47.8	Sakamoto [a]	0.05
	OMM5014	73.8	CO805119	0.02
	OMM5179	95.2	BV211905	0
	OMM1271	116.1	AF470036	0.64
RT22	OMM1023	3.4	AF346681	2.58

	OMM1744	32.8	BV212219	1.87
	Str58CNRS	51.8	U60223	2.23
	OMM1445	68	BV079589	1.43
	Ssa420UOS	92.3	AJ402737	0.98
RT23	OMM1069	0.9	AF375018	0.02
	OMM1097	7	AF352763	0
	Omy1501INRA	33.6	BV681375	0
	OMM1339	55	G73572	0.69
	OMM1190	72.8	AF469975	0.26
	Omy1278INRA	91.2	BV681436	0.43
	Omy1103INRA	116.4	BV681351	1.12
RT24	OmyFGT16TUF	0	Sakamoto [a]	0.01
	OMM1045	9.7	AF346692	0.22
	OMM1211	19.3	AF469995	0.36
	Omy1342INRA	31.5	BV686468	1.34
	Ssa85DU	49.9	U43692	6.79
	Omy4DIAS	66.8	AF090389	8.37
	OMM1322	79.7	G73560	3.5
RT26	Omy1517INRA	21.9	GF111273	0.12
	OMM3000	37.1	G73802	0
	SSOSL32/2	62.5	Z69642	0.01
RT27	Omy29INRA	13.5	Pr009689149.1	6.06
	OMM1131	18.6	AF375032	6.05
	One18/1ASC	24.7	U56718	10.76
	Ocl2UW	44.5	AF028699	0.59
	Omy272/1UoG	46.3	Jackson [d]	0.21
	OMM1070	59.8	AF375019	1.84
	Omy25INRA	101.7	Pr009689147.1	0.84
RT29	OMM1033	0.9	AF375013	9.73
	Ots108SSBI	14.2	AF069680	9.96
	OmyFGT3TUF	39.7	Sakamoto [a]	3.79
	OMM1705	57.4	BV212187	0.03
	Omy21INRA	77.1	Pr009689145.1	1.24
	OMM1187/2	109.9	BV681499	1.85
RT30	Omy108INRA	28.3	Gharbi [e]	0.66
	OMM1238	48.1	AF470018	0.06
RT31	One18/2ASC	12	U56718	0.01
	Omy272/2UoG	21.4	Jackson [d]	0.75
	Omy1006UW	42	AY518329	3.29
	OMM1058	53.2	AF352741	3.49
	OMM1083	54.1	AF352751	5.41
	BHMS129	76	AF256764	13.16
	OMM5164	88.4	BV211874	23.85
	OMM5005	118.7	CO805111	144.95
	Omy1392INRA	119.5	BX861189	153.98

References:

[a] Sakamoto 1997 PhD Thesis, Tokyo Univ. Of Fisheries

[b] O'Connell *et al*. 1997 Can J Fish Aquat Sci 54:1391
[c] McConnell *et al*. 1995 Can J Fish Aquat Sci 52:1863-1872
[d] Jackson *et al*. 1998 Heredity 80:143
[e] Gharbi *et al*. 2006 Genetics 172(4): 2405–2419
Position: marker positions are given according to Guyomard *et al*. 2012.
χ^2: comparison of allele frequencies (R *vs.* S grandparental allele) in the two population tails (early dead & survivors) at each locus.
