# Supplementary material for: Resistance to a Rhabdovirus (VHSV) in Rainbow Trout: Identification of a Major QTL Related to Innate Mechanisms
Source: PLoS One. 2013 Feb 4;8(2):e55302. doi: 10.1371/journal.pone.0055302 (PMC3563530; doi:10.1371/journal.pone.0055302)
Supplement: Table S2 — Markers used for detection of survival-associated QTL and results of association tests for family DH-F00. (PDF) [file pone.0055302.s002.pdf]

TABLE S2

List of microsatellite markers used for detection of survival-associated QTL (DH-F00)

Linkage Group	Marker	Position	Accession number	χ^2
RT1	OmyFGT19/1TUF	7.6	Sakamoto [a]	0.82
	Omy1200INRA	24.2	BV681488	0.18
	Omy17INRA	54.2	Pr009689143.1	0.02
RT2	Str1BFRO/1	34.9	U90327	0.01
	Omy1197/1INRA	17.3	BV681489	0.03
	OMM1200	111.2	AF469985	0.01
	OMM1218/1	115.4	AF469991	0.04
RT3	Omi161TUF	12.3	AB105853	2.24
	OMM1230	37.2	AF470010	0.27
	OGO2/1UW	72.1	AF007827	0.27
	Omy1137INRA	91.7	BV681523	0.1
RT4_25	OMM1193	15.6	AF469978	0.41
	OMM1389	107	BV078075	0.47
	OMM1054	132.8	AF352739	0
RT5	OMM1728	26.4	BV212208	1.94
	OMM1032	60.3	AF352737	0.03
RT6	OMM1231	9.2	AF470011	0.07
	Omy1143INRA	36.7	BV681517	3.38
	OMM1780	63.5	BV212247	1.78
	OMM1454	72.7	BV079598	1.61
	Cocl-lav221	109.2	AY453223	0
	Omy16INRA	135.9	Pr009689142.1	0.04
RT7	Ogo4UW	3.3	AF009796	0.02
	Omy207UoG	29.7	O'Connell [b]	0.36
	OMM1087	57.8	AF352756	1.84
	One1/2ASC	96.9	U56699	0.34
RT8	OMM1075	0	AF352746	0.62
	Omy1390INRA	7	BV681569	5.58
	OmyUW1198	25.3	AY505310	10.03
	OMM1792	37.1	BV212253	13.03
	Omy18INRA	59.4	Pr009689144.1	10.8
	OMM1009	66.3	AF346671	11.6
	Omy1042INRA	88.3	BV681405	2.06
RT9	OMM1218/2	4.1	AF470000	4.72
	OMM1200/2	4.1	AF469985	6.74
	OmyFGT18/2TUF	5.2	Sakamoto [a]	7.88
	OMM1161	15	AY039643	0.84
	OmyFGT28TUF	24.5	Sakamoto [a]	1.25
	OMM1711	39.5	BV212192	2.35
	OMM1450	58.7	BV079594	1.29
	OMM1130	84.6	AF375031	4.01
	OMM1096	98.8	AF352762	3.74

RT10	OMM1179	0	AF469966	0
	One10ASC	17.8	U56710	0
	OmyRGT2TUF	37.2	AB087587	0.04
	OMM1753	67.7	BV212227	0.17
	OMM5186	85.5	BV212286	0.74
	OMM1202/1	104.3	AF469987	2.93
RT11	Ocl1UW	12.4	AF028694	0.07
	Omy13DIAS	28.5	AF239030	2.92
	Omy1134INRA	41	CR376472	3.12
	OMM1004	52.9	AF375008	3.47
RT12	OMM5233	15.7	CA349039	2.55
	OmyFGT4TUF	36.6	Sakamoto [a]	1.75
	OMM1030	45.4	AF375012	2.3
	OMM3059	78	BV078095	0.01
	OMM1381	95.1	BV078067	0
RT13	OMM1216	6.1	AF469998	0.07
	OmyRGT46TUF	50.4	AB087612	0
RT14	OMM1241	1.7	AF470021	0.45
	Omy1214INRA	23.6	BV681478	1.92
	SSa14DU	54.1	McConnell [c]	2.63
	Omy1374INRA	72.5	BV681404	1.43
	Omy120/2INRA	100.7	Pr009689154.1	1.07
RT15	OMM1455	17.2	BV079599	0.11
	Ots1BML	53.2	AF107029	0.15
	Str11INRA	83.6	Gharbi [d]	1.25
RT16	OMM1101	8.6	AF352765	0.87
	Omy1038INRA	23.1	BV681522	1.5
	Omy1227INRA	44.5	BV681503	7.51
	OMM1013	59.5	AF346674	3.82
	Omy77DU	90.7	Morris [e]	3.15
	OMM1403	109.2	BV078088	1.44
RT17	OMM1076	15.6	AF352747	0.98
	OtsG422	49.2	AF393197	0.85
	Omy1376INRA	66.4	BV681462	2.74
	OMM1775	87.4	BV212243	0.74
RT18	OMM1384	23.7	BV078070	0.15
	Omy1DIAS	46	U93270	4.76
	OMM1311	50.2	G73551	5.51
	OMM1202/1	60.8	AF469987	0.64
RT19	Ocl8UW	0	AF028697	0.02
	Omi174TUF	20.8	AB105854	0.1
	OMM1025	40.2	AF346682	1.51
	OMM1313	60.7	G73553	0.02
	OMM1333	93.6	G73567	3.59
RT20	OMM1050	11.8	AF346694	0.05
	SsaN82LEE	28.4	U86706	1.54
	Omy8DIAS	48.5	AF090390	0.61

	Omi102/1TUF	78.8	AB105844	1.2
	Omy120/1INRA	95.7	Pr009689154.1	1.15
RT21	Omy325UoG	0	O'Connell [b]	0.1
	Omy301UoG	23.4	Jackson [f]	1.37
	OmyFGT2TUF	21.8	Sakamoto [a]	1.55
	Omy2DIAS	47.8	AF039065	5.01
	OMM5014	73.8	CO805119	0.01
	OMM5179	95.2	BV211905	0.01
	OMM1271	116.1	AF470036	0.33
RT22	OMM1023	3.4	AF346681	1.52
	OMM1744	32.8	BV212219	0.09
	Str58CNRS	51.8	U60223	5.73
	OMM1445	68	BV079589	0.75
RT23	OMM1069	0.9	AF375018	4.24
	OMM1459	0.9	BV079603	3.3
	OMM1097	7	AF352763	1.45
	Omy27INRA	30.2	Pr009689148.1	0.27
	OMM1719	54.2	BV212200	0.43
	OMM1190	72.8	AF469975	0.84
	Omy1278INRA	91.2	BV681436	0.25
	OmyFGT23TUF	118.8	Sakamoto [a]	0
RT24	OmyFGT16TUF	0	Sakamoto [a]	3.88
	OMM1045	9.7	AF346692	3.31
	OMM1211	19.3	AF469995	9.79
	Ssa85DU	49.9	U43692	0.69
	OmyPupupyDAL	50.8	Morris [e]	0
	OMM1322	79.7	G73560	0.73
RT26	Omy1321INRA	0	BV681520	3.9
	Omy1517INRA	21.9	GF111273	13.34
	OMM3000	37.1	G73802	7.8
	OmyFGT24TUF	59.1	Sakamoto [a]	3.28
RT27	Omy29INRA	13.5	Pr009689149.1	0
	Str4/1INRA	35.3	Gharbi [d]	0.94
	Ocl2UW	44.5	AF028699	0.72
	Omy272/1UoG	46.3	Jackson [f]	0.53
	OMM1070	59.8	AF375019	1.78
	Omy1264INRA	85.9	BV681587	0
RT29	Ots108SSBI	14.2	AF069680	0.21
	OmyFGT3TUF	39.7	Sakamoto [a]	0.14
	OMM1705	57.4	BV212187	1.55
	Omy21INRA	77.1	Pr009689145.1	5.08
RT30	Omy108INRA	28.3	Gharbi [g]	0.43
	OMM1238	48.1	AF470018	0.11
RT31	OmyFGT8/2TUF	12	Sakamoto [a]	0.52
	Omy272/2UoG	21.4	Jackson [f]	2.09
	OMM1058	53.2	AF352741	0
	OMM1083	54.1	AF352751	0

OMM5164	88.4	BV211874	91.05
OMM1080	97.9	AF352750	112.24
Omy1009INRA	107.4	BV681599	148.33
Omy1136INRA	113	BV681527	167.56
OMM5005	118.7	CO805111	201.43
Omy1392INRA	119.5	BX861189	221.01

References:

[a] Sakamoto 1997 PhD Thesis, Tokyo Univ. Of Fisheries

[b] O'Connell *et al*. 1997 Can J Fish Aquat Sci 54:1391

[c] McConnell *et al*. 1995 Can J Fish Aquat Sci 52:1863-1872

[d] Gharbi and Guyomard, Jouy-en-Josas,INRA

[e] Morris *et al*. 1996 Can J Fish Aquat Sci 53:120

[f] Jackson *et al*. 1998 Heredity 80:143

[g] Gharbi *et al*. 2006 Genetics 172(4): 2405–2419

Position: marker positions are given according to Guyomard *et al*. 2012.

χ^2: comparison of allele frequencies (R *vs.* S grandparental allele) in the two population tails (early dead & survivors) at each locus.
